# Retro-Odontoid Intradural Synovial Cyst Decompression via Endoscopic-Assisted Far-Lateral Approach C1-C2 Hemilaminectomy Without Fusion: The Use of Intracranial Denticulate Ligament as Intraoperative Landmark

**DOI:** 10.7759/cureus.21715

**Published:** 2022-01-29

**Authors:** Michael Fana, Christos Deamont, Khalid Medani, Rehan Manjila, Sandeep Kandregula, Donald Labarge III, Sunil Manjila

**Affiliations:** 1 Field Neurosciences Institute Laboratory for Restorative Neurology, Central Michigan University College of Medicine, Saginaw, USA; 2 Department of Internal Medicine, Ballad Health Norton Community Hospital, Norton, USA; 3 Department of Occupational Medicine, Loma Linda University Medical Center, Loma Linda, USA; 4 Swanson School of Engineering, University of Pittsburgh, Pittsburgh, USA; 5 Department of Neurosurgery, McLaren Bay Region Hospital, Bay City, USA; 6 Department of Diagnostic Imaging, McLaren Bay Region Hospital, Bay City, USA

**Keywords:** endoscopy, intradural extramedullary mass, atlanto-axial cyst, synovial cyst, retro-odontoid cyst

## Abstract

Purely intradural retro-odontoid synovial cysts are rarely reported in neurosurgical literature, particularly in the absence of associated bony erosions. We present the case of a 57-year-old Native American male with a retro-odontoid synovial cyst and a history of chronic refractory neck pain that was adequately decompressed via an endoscopic-assisted far-lateral approach using a C1-2 hemilaminectomy, obviating the vertebral artery (VA) transposition, bony instability, and the need for instrumented bony fusion. The patient presented to our clinic with several months of refractory nuchal and cervical spine pain and crepitation affecting his activities of daily living (ADL). MRI findings revealed an intradural cyst at the level of C2 behind the odontoid process impinging on the medulla and causing early VA displacement. Both stereotactic neuro-navigation and microsurgical visualization aided in the manipulation of the endoscope and attaining the caudocranial working trajectory. The patient remained neurologically non-lateralizing postoperatively, similar to his preoperative status. This article highlights a less invasive surgical exposure with an endoscope-assisted caudocranial trajectory obtained by a limited unilateral hemilaminectomy to achieve the desired outcome.

## Introduction

Retro-odontoid synovial cysts are rare diseases that arise from the tectorial membrane posterior to the dens of the axis, causing high cervical cord compression and myelopathy. These occur more often in elderly patients and in those with inflammatory conditions, such as rheumatoid and psoriatic arthritis [1,2]. There is a lack of clarity regarding the progressive development of these cysts. They may, however, be attributed to arthritic degenerative changes in the atlantoaxial joint over long periods of time, chronic mechanical stress, degenerative calcification, or post-traumatic pseudo-arthrosis of the odontoid process [3]. Given the nature of their appearance on cervical spine imaging and symptomology, the differential diagnosis typically includes cystic meningiomas, chordomas, and ecchordosis physaliphora [4]. They may often present in a similar manner to retro-odontoid pseudotumors, which can also develop at the craniovertebral junction (CVJ) adjacent to the odontoid process. These pseudotumors may cause compression of the cervicomedullary junction and are known to be caused by a variety of pathologies, such as rheumatoid arthritis, trauma, os odontoideum, long-term hemodialysis, amyloidoma, atlantoaxial hypermobility, calcium pyrophosphate, systemic lupus erythematosus, tumors, and spinal disc herniation [5]. Moreover, with synovial cysts, there is diminished articulation at this joint space from damage to cervical ligaments and their attachment sites in the craniocervical junction, which may be the root cause of the growth of the benign cyst [6].

In current clinical practice, imaging modalities such as MRI are unable to effectively differentiate synovial cysts from other cervical spine pathologies, such as a pannus or calcium pyrophosphate deposits. Often, the diagnosis is not confirmed until surgical resection is complete with histologic confirmation, and patients have been reported to typically present with occipital and neck pain, numbness, headaches, and lower cranial nerve deficits [5,7].

Currently, three conventional strategies for the resection of retro-odontoid lesions exist, each with certain drawbacks that must be given consideration: the anterior transoral approach, which carries a higher risk of infections, particularly with transdural access; the endoscopic endonasal approach, which requires specialized neurosurgical training to access the caudal trajectory and carries a high risk of infection after durotomy; the lateral-high cervical approach, which predisposes to the possibility of vertebral artery (VA) mobilization over C1; and the posterolateral/far-lateral approach, which involves complex dissection and risks epidural bleeding and pseudomeningocele formation [8]. In some cases, where surgery is contraindicated for the patient, a percutaneous aspiration is also a viable option. However, this approach has higher chances of cyst reformation and recurrence [9]. Currently, the posterior or posterolateral approach is often preferred for cysts that form at the lateral aspect of the transverse ligament as it offers the shortest trajectory [10].

The intracranial denticulate ligament (i.e., the highest denticulate ligament) can serve as an important landmark in operative approaches at the CVJ. These extensions of the pia-arachnoid layer can facilitate the delineation between the anterior and posterior spinal compartments [11]. This ligament is attached to the dura of the marginal sinus superior to the VA and lies inferior to the spinal accessory nerve. When operating on intradural CVJ or foramen magnum lesions, this ligament can be dissected to allow for manipulation of the spinal cord with minimal retraction or damage [12].

In this report, we present the case of a middle-aged patient with a retro-odontoid cyst associated with a history of chronic refractory neck pain and no evidence of myeloradiculopathy. The cervical spine was decompressed via an endoscopic-assisted far-lateral approach for a C1-2 hemilaminectomy without fusion. The pathology report confirmed a synovial cyst and the patient was neurologically intact postoperatively. Given our patient’s history, we hypothesize the cyst's origin to be ligamentous degeneration. This article demonstrates the surgical trajectory with endoscopic guidance and reviews other surgical approaches for retro-odontoid cysts in the current literature.

## Case presentation

A 57-year-old Native American male presented with the chief complaint of persistent neck pain and crepitation in the upper neck for several months. On examination, the patient showed no signs of radiculopathy or myelopathy. Relevant past medical history included chronic neck pain, possibly as a consequence of a prior automobile accident. The patient had no symptoms of peripheral neuropathy, urinary incontinence, or improvement in neck pain. Examination of his upper and lower extremities demonstrated full strength with intact and equal sensation bilaterally but with diffusely diminished reflexes.

**Figure 1:**
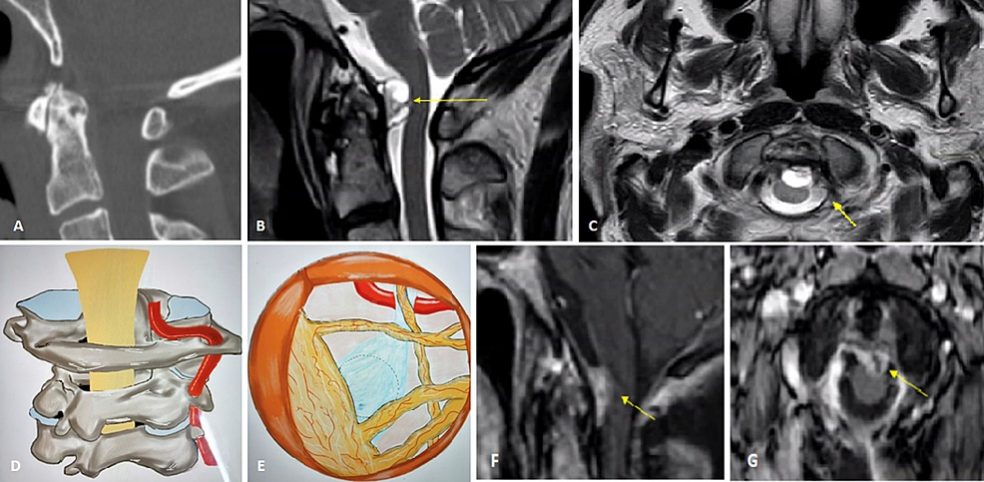
(A) CT neck demonstrating mild focal hyperostosis behind the dens. (B) Sagittal and axial (C) MRI of the head and neck demonstrating spinal stenosis at C1-2 from intradural extramedullary mass (IDEM) (arrow) impinging on the spinal cord at the level of dens. (D, E) Artist's rendering of the location of the retro-odontoid intradural mass. (F) Postoperative sagittal and axial (G) MRI of a resected cystic portion of the tumor and reduced mass effect (arrow) on the brainstem CT: computed tomography; MRI: magnetic resonance imaging

A cervical X-ray taken four years prior to our evaluation had shown arthritic changes at the C1-2 levels with multilevel spondylosis. CT neck at the time had revealed similar changes with moderate degeneration in the cervical spine and multilevel, moderate neuroforaminal stenosis, and mild focal hyperostosis behind the dens (Figure 1A).

MRI of the head and neck also showed spinal stenosis at C1-2 due to an intradural extramedullary mass (IDEM) impinging on the spinal cord at the level of dens (Figures 1B, 1C). This ventral intradural cystic mass was clinically and radiologically followed up over two years, and it was found to have increased in size at interval studies, causing increasing brainstem compression. It was decided to resect the mass via a stereotactic trajectory after positioning with a smaller scalp flap for a far-lateral approach as there was no suboccipital craniotomy. An intraoperative lumbar drain was placed for reducing the postoperative increase in CSF pressure over the dura suture line.

An endoscope-assisted microsurgical cyst decompression from a left far-posterolateral C1-2 hemilaminectomy was then performed without causing any bone instability or VA transposition. After the semicircular opening of the dura mater based on the VA axis and reflection of the dura flap laterally, arachnoid dissection was performed to release CSF. The first denticulate ligament served as an intraoperative landmark, the cutting of which offered simplified maneuvering of the endoscope in the limited lateral intradural space at the CVJ. This unique IDEM at the level of the C2 dens exuded a green-yellow fluid from the mass with partly yellowish, flaky material within the capsule. The tumor wall was noted to be extremely thick, yellow-white, fibrous, and firm. The final histopathology report confirmed a synovial cyst at the retro-odontoid region.

**Figure 2:**
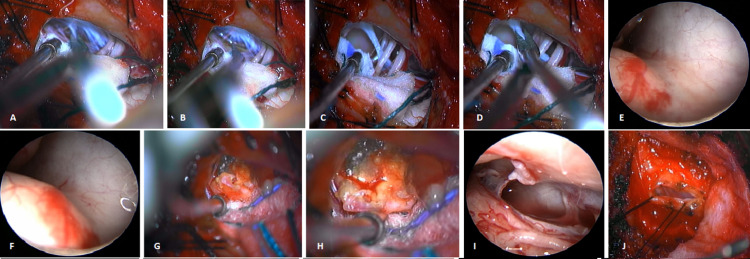
Intradural exposure demonstrated after C1-2 hemilaminectomy, with a close-up endoscopic view of the dura attachment after sacrificing the denticulate ligament

The extent of dura exposure after C1-2 hemilaminectomy, limited durotomy, and close-up endoscopic view of the dura attachment after sacrificing the denticulate ligament are demonstrated as a pictorial essay (Figures 1D, 1E, and Figures 2A-2J).

The greenish-yellow cyst fluid with yellow, flaky contents and the thick, fibrotic capsule with surface hypervascularity of the cyst merging with the dura is shown in Figures 2G, 2H. The patient recovered well with a short hospital stay of three days and remained neurologically intact at baseline on follow-up. Postoperative MRI confirmed the removal of the cystic portion of the tumor and reduced mass effect on the brainstem (Figures 1F, 1G).

## Discussion

Exclusive dura-based ventral extramedullary cysts of the cervical spine are rare and may occur without bone erosion or neurological deficits. The differential diagnosis of such lesions in the ventral skull base may include cystic meningiomas, chordomas, pseudotumors, rheumatoid pannus, ecchordosis physaliphora, and synovial cysts of C1-2 [4]. In our patient, we described a rare case with an interval increase in size and mass effect from this IDEM, which was operated successfully with endoscopic assistance.

The pathogenesis of cervical intradural synovial cysts may be multifactorial. It is likely that atlantoaxial instability causing subluxation or chronic micromotion of the C1-2 vertebrae contributes significantly to its development [13]. Consequently, this instability can lead to progressive refractory neck pain and compressive cervical myelopathy, which becomes potentially disabling if left untreated. This destabilization appears to come from chronic stress to the facet and uncovertebral joints [14]. We concur and suggest that chronic focal irritation and inflammation of the retro-odontoid ligaments contribute to cyst formation by way of abnormal movement of the atlas on the dens. Such movements elicit torsional stress and exacerbate cervical motions, particularly in extension of the cervical spine. This is validated by the inhibition of stress and reduction of inflammation to these joints after C1-2 fusions. Similarly, soft tissue hypertrophy of the retro-odontoid region has also been found to reduce after fusion of C1-2 [15].

**Figure 3:**
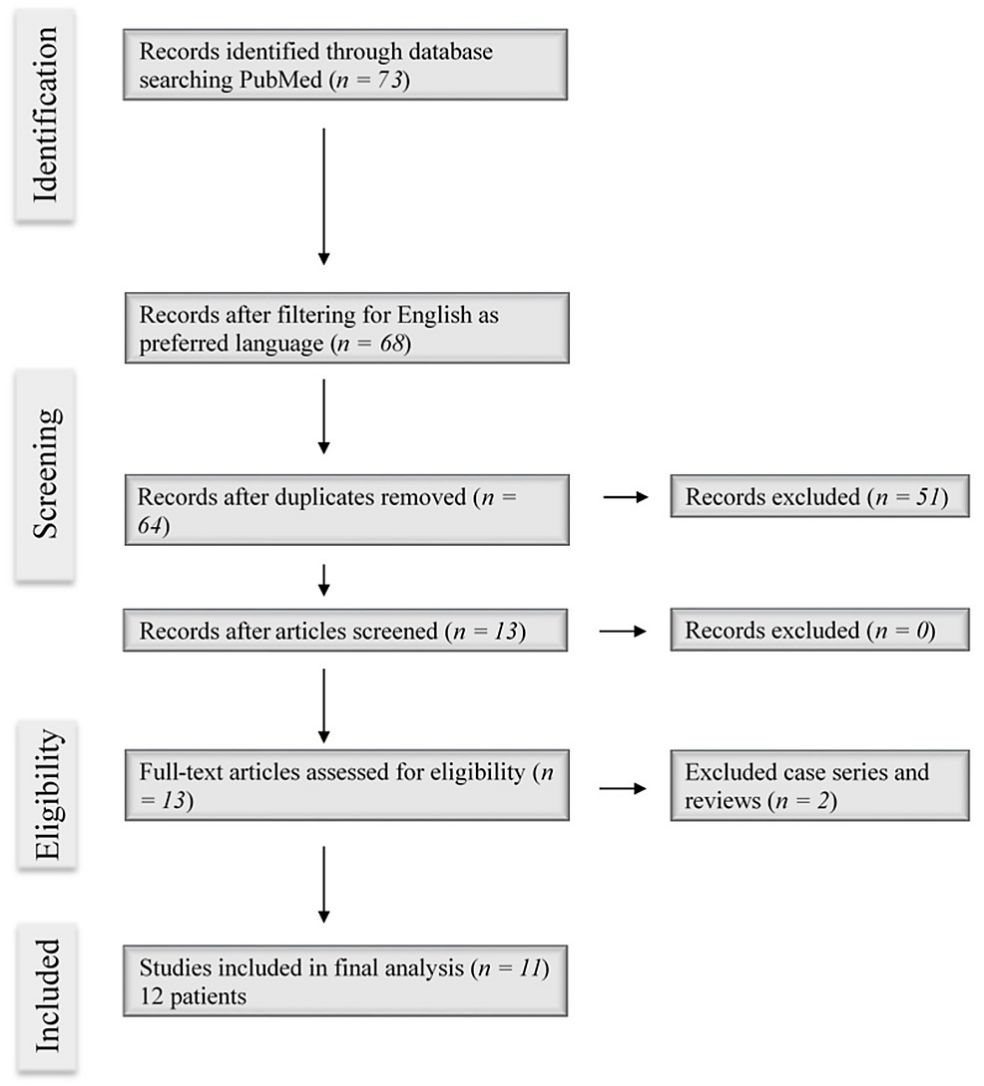
PRISMA flowchart showing the search strategy for current literature and article selection for analysis Search function: "intradural atlantoaxial cyst", "retro-odontoid cyst", and "odontoid cyst" yielded 73 results initially PRISMA: Preferred Reporting Items for Systematic Reviews and Meta-Analysis

Retro-odontoid cysts are commonly removed surgically and, in some cases, non-surgically with percutaneous aspiration for non-surgical candidates. However, there are few case reports of synovial cysts in the atlantoaxial region in the literature to propose a definitive surgical approach. Our literature review of the PubMed database for "intradural atlantoaxial cyst" and "odontoid cyst" yielded 73 results. After the screening of results by removing the duplicates, excluding case series and reviews, removing case reports involving calcium pyrophosphate deposition, and filtering for English as the preferred language, 10 case reports involving 11 patients were identified (Figure 3). The salient details of the operation in each case report are highlighted in Table 1 [2,3,6,9,14,16-18,20,21]. Even though each approach varied according to patient profile, symptomatology, and tumor location, there is consistency in the resolution of the synovial cyst in most cases, suggesting the possibility of multiple approaches to intervention.

**Table 1:** Pertinent case reports regarding the surgical and non-surgical management approach toward the resection of retro-odontoid cysts CT: computed tomography; MRI: magnetic resonance imaging

Case report	Patient age and sex	Symptoms	Procedure	Postoperative outcome
Ikegami et al. [16]	52; female	Neck pain, numbness, and hypesthesia in hands	Lateral atlantoaxial joint puncture and arthrography	Improvement in numbness and hypesthesia of hands. Reduction in cyst size at one-month follow-up with complete disappearance at five months
Le Pape et al. [21]	51; female	Cervical pain, paresthesia in the right upper limb, pyramidal syndrome	C1-C2 arthrodesis	Paresthesia regression and motor recovery on day three postop. Neurological recovery by three months with some mild cervical pain. MRI confirmed the disappearance of cyst
Lin et al. [14]	64; female	Neck pain, hand and foot paresthesia	Posterior C1 and partial C2 laminectomy, no cyst resection	Complete cyst regression and symptom-free at one-year follow-up
Madhavan et al. [2]	70; male	Worsening gait, recurrent falls, loss of balance, bladder incontinence, and pain in the occipital region	Posterior suboccipital craniectomy and C1 and partial C2 laminectomy	Improvement in overall symptoms at one month with no symptoms at 19 months
Madhavan et al. [2]	74; female	Right-sided neck pain with limited extension and lateral rotation of the neck	Posterior suboccipital craniectomy and C1 and partial C2 laminectomy	Minimal pain with good healing at two-week follow-up with complete resolution of cyst and symptoms at seven months
Meng and Liu [17]	50; male	Cervical myelopathy	Posterior reduction and occipitocervical fusion without cyst resection	Follow-up in five months showed cyst regression with the reduction in atlantoaxial dislocation and opening of cervicomedullary angle to 140^o^
Ogata et al. [18]	58; female	Neck pain, numbness in hands and limbs, hyperreflexia, loss of grip strength, and bladder-rectal disorders	Posterior fixation of C1-C3 without cystic mass resection	Fusion between C1 and C3 appreciated at three months postop with complete resolution of the cystic mass and symptoms
Ohnishi et al. [3]	70; male	Right upper extremity myelopathy, increased deep tendon reflexes in right upper and bilateral lower extremities	Right-sided anterolateral approach with resection of cystic mass without fusion	Improvement in gait disturbance and myelopathy after two weeks without cervical neck pain. No atlantoaxial instability after a three-year follow-up
Takeuchi et al. [6]	76; male	Neck pain, hand and foot paresthesia, dysarthria, dysphagia	Left posterolateral approach with suboccipital craniotomy and C1 hemilaminectomy, atlantoaxial fixation at C1-C2	Immediate improvement in dysarthria and dysphagia. Mass reduction at three months. Full recovery at 12 months
Velán et al. [9]	92; female	Intense cervical pain, progressive spastic quadriparesis	CT-guided percutaneous aspiration of the cyst	Immediate relief of pain and improvement in quadriparesis. Recurrence of the cyst at 1.5 years
Sameshima et al. [20]	69; male	Paralysis and sensory disturbance of the right arm and leg	Transdural approach with partial transcondylectomy and C1 hemilaminectomy	Immediate relief of weakness of the right arm and leg after surgery. Disappearance of cyst on MRI
Present Case	57; male	Neck and right shoulder pain with crepitation, hyporeflexia of extremities bilaterally	Far-posterolateral endoscope-assisted microsurgical resection with left hemilaminectomy of C1 and C2 without fusion	Full neurological recovery with a short hospital stay of three days. Resolution of pain and neurologically intact on follow-up

Our patient did not present with myelopathy or lower cranial neuropathy. Moreover, there was an absence of any major bony destruction on CT scan or abnormal movement on dynamic imaging of the high cervical spine at the C1-2 level. Therefore, we opted for an endoscope-assisted far-lateral stereotaxic approach. We did not drill down the upper C1 lamina and preserved the VA without transposition. Furthermore, we did not create any bony instability that would mandate posterior cervical fusion. The endoscopic visualization offered additional help in this surgery to closely observe the dura attachment (superior and lateral) without sacrificing bone that would cause C1-2 instability or compression on the high cervical spinal cord. The denticulate ligament served as an anatomical landmark and its sacrifice created room for manipulating surgical instruments, including a rigid endoscope through such a small durotomy. Moreover, this approach eliminated the prospect of injury to the vertebral artery and was preferable over an anterior approach given the higher risk of infection, spinal fluid leak, longer operative time, and need for posterior instrument stabilization [2].

Several of the case reports documented the use of a less invasive approach for the treatment of a retro-odontoid cyst. For instance, a lateral atlantoaxial joint puncture and arthrography is one approach that is minimally invasive and provides therapeutic resolution of the cyst and relative neurological symptomatic improvement over months [16]. Another is a CT-guided percutaneous approach for cyst volume drainage and reduction [9]. However, in the latter case, the patient required readmission for drainage once more as the tumor had regrown during the initial postoperative period. Evidently, while offering a less invasive approach with rapid relief of symptoms for patients who are not ideal candidates for surgery is an option, aspiration may not definitively treat the cyst and prevent recurrence or regrowth.

Madhaven et al. (2018) have reported cases of three patients, two of which were retro-odontoid cysts that completely resolved with a posterior suboccipital craniectomy and laminectomy. Interestingly, Lin et al. (2014), Meng and Liu (2016), and Ogata et al. (2007) also approached a synovial cyst with posterior laminectomy, occipitocervical fusion, and posterior fixation, respectively, but without surgical resection due to technical challenges. Nonetheless, all of them reported resolution of the patients' symptoms along with regression of the cysts over several months [14,17,18]. Meanwhile, an anterior approach via the sternocleidomastoid muscle can permit access to the dura for cyst resection [3].

Takeuchi et al. (2011) also approached a retro-odontoid cyst with a posterolateral approach with C1-2 fixation but did not completely resect the mass, which was adhered tightly to the brainstem. Nevertheless, follow-up in three months showed mass reduction without any neurological symptoms [6]. Evidently, the benign nature of the cystic mass may allow for partial or near-complete resection without requiring full removal for clinical symptom improvement. The mass resected did not have a synovial cell lining as seen in ganglion cysts. Curiously, however, it contained necrotic fibrocartilage with fibroconnective and granulation tissue without mucin deposits [6]. This suggests that the cyst consisted of degenerative ligamentous tissue instead of mucinous material often seen in synovial or ganglion cysts. Ganglion cysts, much like synovial cysts, are benign lesions that rarely develop in the spine. These cysts derive from joint capsules or tendon sheaths of limb joints, but in the retro-odontoid space, they may arise from cruciate or transverse ligament degeneration. Over time, and with progressively rising intra-cystic pressures, the lining of the cyst capsule disappears and develops into a ganglion cyst. Synovial cysts, on the other hand, retain a film of tissue around the cyst as synovial cells begin to proliferate outside the capsule space [19]. Likewise, Sameshima et al. (2013) resected a cyst in the retro-odontoid epidural space via a transdural approach for transcondylectomy and C1 hemilaminectomy, resulting in the complete resolution of symptoms [20]. The cyst wall consisted of fibrous connective tissue, consistent with a synovial cyst, but was continuous with the transverse ligament [20]. Clearly, the possibility that such retro-odontoid cysts develop as a consequence of prolonged wear and tear on cervical ligaments, causing necrosis of the tissue with microbleeds, must be explored further. Nevertheless, while the specific surgical approach to cyst resection can be patient- and surgeon-dependent, we have for the time being reported a rare case involving limited dissection of tissue planes, bone removal, and vessel transposition, largely accomplished thanks to endoscopic assistance and stereotactic neuro-navigation.

## Conclusions

Synovial cysts of the spine, in particular of the atlantoaxial joint space, are exceptionally rare occurrences found in elderly patients and can in some instances cause myelopathy. Symptomatic retro-odontoid cysts are often treated surgically using either an anterior transoral, endoscopic endonasal, far-lateral high cervical, or posterolateral approach depending on the individual patient profile. We presented a patient with a rare case of a retro-odontoid intradural synovial cyst treated with an endoscope-assisted far-posterolateral surgical approach without needing vertebral artery transposition or instrumented C1-2 fusion. The anatomical marker used in this approach (i.e., the highest denticulate ligament) is discussed along with the utilization of a rostrocaudal surgical trajectory given the endoscope assistance. The role of Stealth neuro-navigation and the endoscopic-assisted "fish-eye" view of the lesion are illustrated in the article.
